# Clinical characteristics and early recognition of gastroptosis: a propensity score-matching analysis

**DOI:** 10.3389/fmed.2026.1916142

**Published:** 2026-08-19

**Authors:** Jiyuan Zhang, Hang Yin, Tianyu Liu, Chunzhao Yu

**Affiliations:** Department of General Surgery, Sir Run Run Hospital, Nanjing Medical University, Nanjing, Jiangsu, China

**Keywords:** aortomesenteric angle, gastric motility index, gastroptosis, propensity score matching, risk factors

## Abstract

**Background:**

While some surgeons have reported that gastroptosis is associated with relaxation of abdominal wall muscles and ligaments, the influencing factors related to gastroptosis still remain incompletely understood.

**Aim:**

To analyze the clinical characteristics of gastroptosis and identify independent associated factors for early recognition.

**Methods:**

A total of 188 patients with confirmed gastroptosis and 376 non-gastroptosis controls were initially enrolled. The degree of gastroptosis, gastric motility parameters, and the aortomesenteric angle were evaluated via oral gastric contrast-enhanced ultrasound (OGUS) in a specific fasted, sitting position. After balancing confounding factors using 1:1 propensity score matching (PSM), inter-group clinical variables were investigated via univariate analysis; logistic regression was then utilized to identify predictors of gastroptosis. Finally, a receiver operating characteristic (ROC) curve was constructed to determine the optimal predictors of gastroptosis.

**Results:**

Univariate analysis showed significant differences between the two groups in body mass index (BMI), gastric motility index (GMI), aortomesenteric angle, duodenal reflux, and bloating. Additionally, GMI and aortomesenteric angle significantly varied across different gastroptosis grades. Logistic regression identified lower BMI, lower GMI, and a smaller aortomesenteric angle as independent predictors for gastroptosis. The combined diagnostic model of these three factors demonstrated a strong predictive value, with an area under curve (AUC) of 0.909, yielding a sensitivity of 84% and a specificity of 80%.

**Conclusion:**

Lower BMI, reduced GMI, and a smaller aortomesenteric angle serve as primary independent predictors for gastroptosis, whereas bloating, positive duodenal reflux as secondary clinical indicators. Recognizing these primary and secondary features allows practitioners to stratify high-probability patients early, significantly reducing misdiagnosis, enabling timely, targeted management to prevent severe gastroptosis and ultimately optimizing patient clinical outcomes.

## 1 Introduction

Gastroptosis is an anatomical disorder characterized by downward displacement of the stomach toward the pelvic cavity, resulting from decreased intra-abdominal pressure or laxity of the ligaments, mesentery, and muscles that maintain the normal gastric position (1). However, no clear epidemiological data on its incidence are currently available. A single screening conducted in China in 2013 reported an incidence of 9.80% (2). While current literature has explored the correlations of gastroptosis with bile reflux, gastroesophageal reflux, and gender (3–6), the impact of BMI, GMI, and other potential determinants remains poorly understood.

Gastroptosis predominantly affects young and middle-aged adults, typically between 20 and 40 years of age (7). Clinically, the primary therapeutic goal for these patients has shifted from anatomical correction to the relief of functional symptoms (8). Furthermore, BMI is closely linked to this condition; a low BMI reflects the depletion of visceral fat depots, which deprives the stomach of essential structural support and precipitates downward displacement. Patients with gastroptosis often present with multiple gastrointestinal symptoms, including dyspepsia, nausea, vomiting, belching, and abdominal discomfort (8). Its diagnosis relies primarily on upper gastrointestinal (GI) series, abdominal computed tomography (CT), and gastroscopy. Because the condition is relatively insidious, the nonspecific clinical manifestations, limited public awareness, and frequent confusion with disorders such as gastritis, esophagitis, and dyspepsia often lead to a prolonged lack of effective diagnosis and treatment, resulting in a marked decline in quality of life. Therefore, a more detailed analysis of the clinical characteristics of gastroptosis, alongside the exploration of its risk factors and the strength of their association with disease onset, is crucial. These insights are essential for early identification and diagnosis, as well as for preventing clinical progression or severe secondary complications, such as malnutrition and gastric outlet obstruction, in high-risk patients (6, 9, 10).

The OGUS offers non-invasive and portable advantages in visualizing gastric anatomy, wall lesions, and motility dynamics (11, 12). Consequently, it serves as a valuable adjunct to endoscopy and upper GI series. Although not yet established as the primary diagnostic tool for gastroptosis, OGUS has demonstrated diagnostic efficacy comparable to upper GI barium radiography (12). Notably, because it avoids radiation and contrast allergen exposure, OGUS is highly suitable for pregnant women, pediatric patients, and individuals with contrast allergies, thereby broadening screening coverage for potential gastroptosis cases. To leverage these advantages, we utilized OGUS to evaluate the severity of gastroptosis and gastric motility alterations in patients previously diagnosed via upper GI series, aiming to provide a more comprehensive characterization of clinical and structural abnormalities in this population.

This article retrospectively analyzes case data of patients with and without gastroptosis admitted to Sir Run Run Hospital Affiliated to Nanjing Medical University from January 2020 to December 2025 as the disease group and control group, respectively. Using PSM to balance confounding covariates between the two groups, we aim to explore the clinical characteristics of gastroptosis, screen for influencing factors, and provide insights for early identification, diagnosis, and prevention.

## 2 Materials and methods

### 2.1 Clinical data

A retrospective analysis was conducted on the clinical records of patients diagnosed with gastroptosis during hospitalization at the Sir Run Run Hospital Affiliated to Nanjing Medical University from January 2020 to December 2025, and we collected baseline information including age, sex, height, and weight. Patients who were not diagnosed with gastroptosis during the same hospitalization period were enrolled as the control group. All the patients who met the following recruitment criteria were enrolled both the group: (1) complete clinical data, including medical history, symptoms, Gastroesophageal reflux disease questionnaire (GerdQ) score, and imaging findings (2) first-time diagnosis and treatment at our hospital. The exclusion criteria for both the group included: (1) history of major gastrointestinal surgery such as pancreaticoduodenectomy or subtotal gastrectomy, (2) presence of severe functional impairment of vital organs, (3) concomitant severe gastrointestinal diseases such as gastric cancer, gastric ulcer, pyloric obstruction and intestinal obstruction, (4) consumptive diseases (e.g., hyperthyroidism, cirrhosis, chronic enteritis, tuberculosis), (5) pregnancy or lactation, (6) incomplete clinical data.

### 2.2 OGUS evaluation

**Figure 1:**
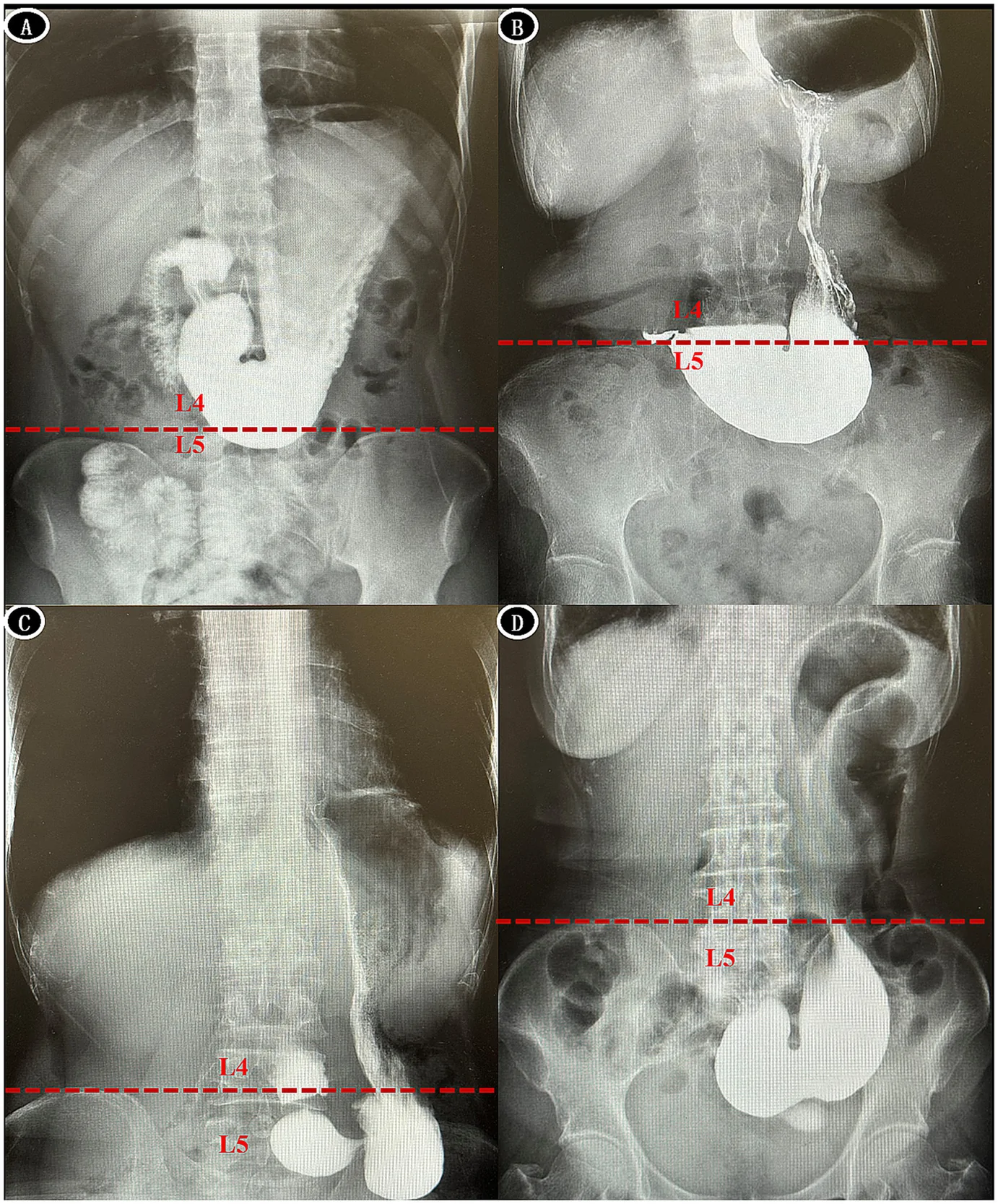
Different grades of gastroptosis in upper GI series: **(A)** normal hook-like stomach, **(B)** grade I (between the L4 ~ L5), **(C)** grade II (L5), and **(D)** grade III (at the upper margin of the sacrum).

All the patients with gastroptosis were diagnosed through upper GI series. According to the diagnostic criteria established by the Japanese Society of Radiology, gastroptosis is diagnosed when the angular incisure of gastric lesser curvature lies ≥1 cm below the iliac crest line (13). The severity of gastroptosis is graded based on the position of the gastric lesser curvature relative to the lumbar vertebrae: grade I (between the L4 ~ L5), grade II (L5), and grade III (at the upper margin of the sacrum) (14, 15) (Figure 1).

Subsequently, we performed OGUS on patients previously diagnosed with gastroptosis. To establish a consistent baseline, historical imaging reports obtained prior to this study were excluded from the analysis. Instead, all participants underwent an identical, newly administered diagnostic protocol performed by the same attending physician to ensure high inter-observer reliability. Prior to the examination, patients were fasted for 8 to 10 h and restricted from fluid intake for 4 h. During the process, patients were maintained in a seated position (16). Following the oral administration of approximately 500 mL of the ultrasound contrast agent, OGUS was performed to evaluate the following parameters: gastric body position, fluid transit across the gastric antrum, pyloric valve, and proximal duodenum, as well as the aortomesenteric angle (17, 18). Owing to the lack of unified diagnostic standards for gastroptosis in OGUS imaging, we graded gastroptosis severity based on the upper GI series criteria (19). Specifically, severity was classified according to the distance between the incisura angularis of the lesser curvature and the interiliac line: mild (<5 cm), moderate (5 ~ 8 cm), and severe (>8 cm). Given the absence of a universally accepted sampling protocol for the gastric antral contraction index, we referred an established methodology from previous literature to evaluate gastric motility via ultrasound (20, 21). Specifically, we recorded the total number of contractions over a 6-min observational window. To facilitate calculations and align with conventional indices, the contraction frequency was subsequently parameterized as the number of contractions per 2-min interval (22). Simultaneously, the maximum diastolic area (*S*_dia_) and maximum systolic area (*S*_sys_) of the gastric antrum were measured three consecutive times, and the average values were calculated. The amplitude of gastric antral contraction was derived as (*S*_dia_ – *S*_sys_)/*S*_dia_. The GMI was calculated as the product of contraction frequency and amplitude (22). Gastric motility was classified based on GMI values as poor (GMI ≤ 0.4), decreased (0.4<GMI <0.8), or good (GMI ≥ 0.8) (22). Subsequently, the pylorus was assessed for ulcers and reflux, and its inner diameter was measured along the long-axis section. Duodenal reflux was also monitored; patients exhibiting a reflux duration of ≥3 s or a reflux frequency of ≥3 times within a 5-min period were diagnosed with duodenal reflux (23, 24).

Previously, numerous studies have reported complications, such as gastroparesis and gastric dilation, caused by superior mesenteric artery syndrome (SMAS). We hypothesized that chronic duodenal compression by the SMAS may be associated with the development of gastroptosis. Therefore, we utilized ultrasonography to compare the aortomesenteric angle between the two groups to explore its correlation with gastroptosis (25–27). Initially, a routine fasting ultrasound examination was performed, followed by a multi-planar scan after the oral administration of a gastric ultrasound contrast agent. Conventional ultrasound was used to measure the aortomesenteric angle in the longitudinal section. Following contrast administration, a transverse scan was performed to visualize the longitudinal section of the duodenum, alongside the transverse sections of the abdominal aorta and SMAS. Intestinal morphology, peristalsis, and contrast transit, as well as the presence of stasis or retrograde peristalsis proximal and distal to the aortomesenteric angle, were systematically evaluated (27).

### 2.3 Gastrointestinal symptoms evaluation

To precisely elucidate the relationship between gastroptosis and GERD without the confounding attenuation inherent in composite scores, we specifically isolated items Q1 (heartburn) and Q2 (regurgitation) from the GerdQ score. Each core symptom was independently evaluated using a standardized 4-point Likert scale: none (0), 1 day (1), 2 ~ 3 days (2), 4 ~ 7 days (3). This strategy is robustly supported by evidence indicating that Q1 and Q2 possess the highest diagnostic specificity and weights within the GerdQ score, whereas total scores can be easily confounded by concurrent gastric motility lag or non-specific dyspeptic traits, thereby providing a clearer mechanistic perspective on how anatomical gastric displacement influences the severity of gastroesophageal reflux (28, 29). Concurrently, to evaluate the broader impact of general gastrointestinal manifestations, other symptoms such as bloating, belching, and inappetence were classified as positive if they occurred at least twice weekly over the preceding 3 months, with sufficient severity to induce noticeable discomfort or impair daily activities. Conversely, a negative status was defined as either the complete absence of the symptom or its negligible presentation (fewer than two episodes per week) without causing clinical distress (30, 31).

### 2.4 Statistical analysis

Given the limited number of previous studies on factors associated with gastroptosis, only a few reports have suggested a higher prevalence in female and younger patients (7, 32). To balance potential confounders between groups and minimize the influence of unknown variables that may be associated with gastroptosis, we used pre-matching variables that showed significant differences (age and sex) as independent variables and the diagnosis of gastroptosis as the dependent variable. A 1:1 PSM was performed using SPSS 29.0, with a matching caliper of 0.01, between 188 cases in the study group and 376 cases in the control group. Propensity scores were calculated for each sample, resulting in a case group and a control group with satisfactory inter-group balance after PSM.

Statistical analyses were performed using SPSS 29.0 and R 4.3.1. For quantitative data, the Kolmogorov–Smirnov test was applied to assess normality. Data following a normal distribution were expressed as mean ± SD ( $\bar{X}$ ± S), whereas non-normally distributed data were presented as median with interquartile range [*M* (Q1, Q3)]. Comparisons between two groups were conducted using the independent samples t-test for normally distributed data or the Mann–Whitney *U* test for non-normally distributed data. Comparisons among multiple groups were performed using the Kruskal–Wallis test. Categorical variables were expressed as counts (%), and group comparisons were carried out using the *χ*^2^ test or Fisher’s exact test, as appropriate. Variables that showed statistical significance in univariate analysis were entered into multivariate logistic regression analysis. A predictive model was then constructed using the statistically significant variables. ROC curve was plotted, and the AUC was calculated to evaluate the relative importance of different variables in model prediction. A two-sided *p* < 0.05 was considered statistically significant.

### 2.5 Ethical statement

The Ethics Committee of Sir Run Run Hospital of Nanjing Medical University approved this retrospective study (approval number: 2024-SR-067) and waived informed consent due to patient data anonymization.

## 3 Results

### 3.1 Patients’ demographic and clinical characteristics

**Figure 2:**
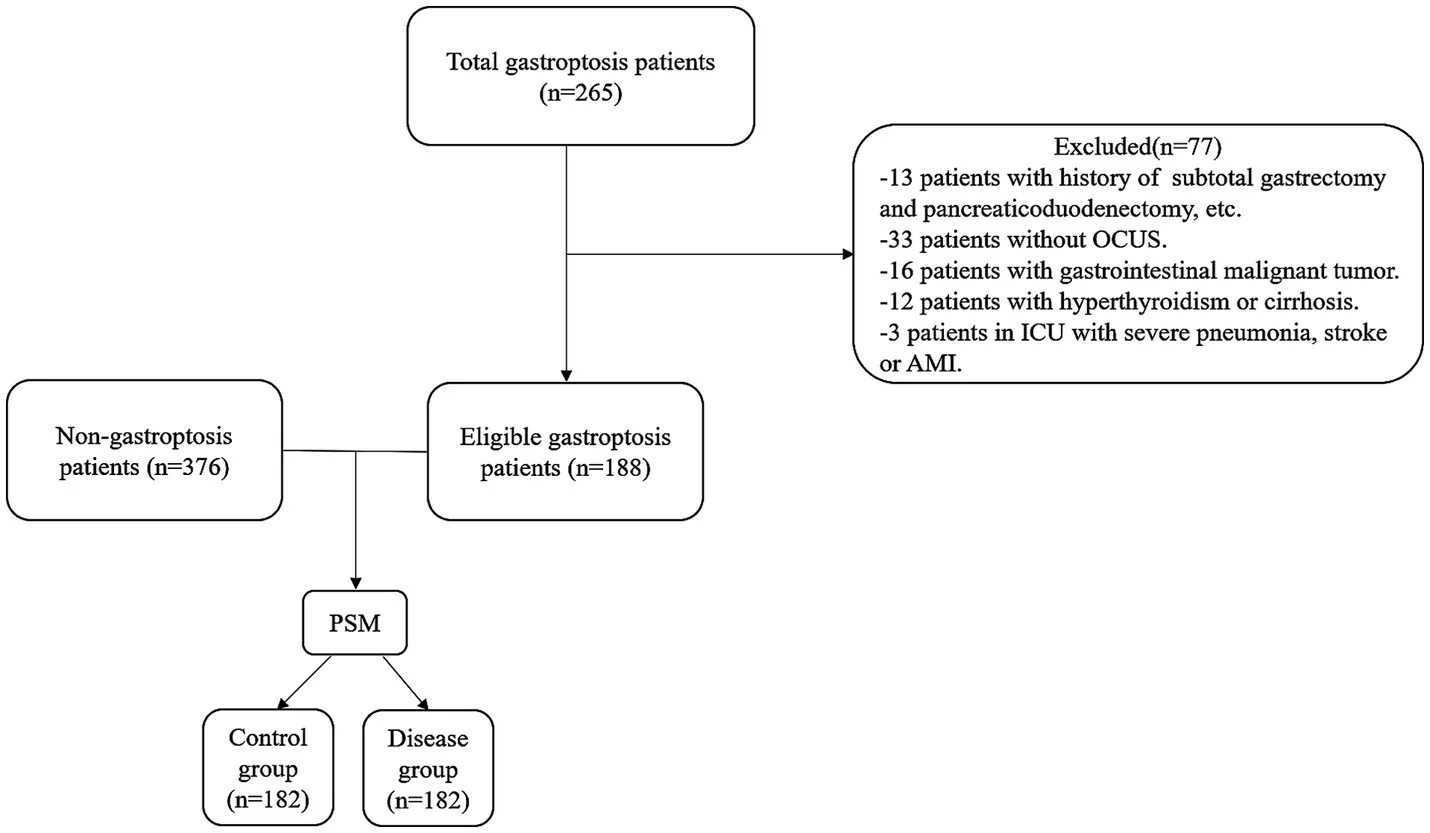
Flowchart for screening eligible patients.

Initially, 265 patients diagnosed with gastroptosis were identified. After applying the exclusion criteria, 188 patients were ultimately included in the disease group (Figure 2). Using a case-to-control ratio of 1:2, two investigators independently and randomly selected 376 patients without gastroptosis who were hospitalized for other benign digestive system diseases during the same period to serve as the control group, yielding a total of 564 participants. After propensity score matching, 182 pairs were successfully matched. Following matching, there were no statistically significant differences in sex or age between the two groups (*p* > 0.05) (Table 1).

**Table 1:** Comparison of basic information between two groups of patients before and after matching.

Variables	Before matching		Test statistic	*P*	After matching		Test Statistic	*P*
	Control group (*n* = 376)	Disease group (*n* = 188)			Control group (*n* = 182)	Disease group (*n* = 182)		
Gender, *n* (%)			14.056^a^	<0.001			0.995^a^	0.188
Male	176(46.80)	57(30.32)			66(36.26)	57(31.32)		
Female	200(53.20)	131(69.68)			116(63.74)	125(68.68)		
Age, *M* (Q₁, Q₃)	47.0(33.3,63.0)	43.5(31.0,57.0)	-2.861^b^	0.004	41.0(30.0,53.3)	44.0(31.0,57.3)	−1.037 ^b^	0.300

^a^Chi-square Test, ^b^Mann–Whitney *U* test.

### 3.2 Univariate analysis and binary logistic regression analysis

Univariate analysis revealed statistically significant differences between the two groups after matching in terms of BMI, GMI, aortomesenteric angle, duodenal reflux, and upper abdominal distension (*p* < 0.05) (Table 2).

**Table 2:** Univariate analysis of clinical characteristics of two groups of patients.

Variables	Control group (*n* = 182)	Disease group (*n* = 182)	Test statistic	*P*
BMI, *M* (Q₁, Q₃)	23.1(21.7,24.1)	20.0(18.3,20.0)	−8.612^b^	<0.001
GMI, *M* (Q₁, Q₃)	2.88(2.55,4.30)	2.40(1.30,2.70)	−10.348^b^	<0.001
Aortomesenteric angle, *M* (Q₁, Q₃)	29(23,33)	25(22,26)	−7.282^b^	<0.001
GERD^c^, *n* (%)	36(19.9)	43(23.6)	0.792^a^	0.223
Duodenal reflux, *n* (%)	46(25.3)	83(45.6)	16.438^a^	<0.001
Cholecystectomy, *n* (%)	55(30.2)	58(31.9)	1.116^a^	0.410
*Hp^d^* infection, *n* (%)	62(34.1)	75(41.2)	1.978^a^	0.097
Diabetes, *n* (%)	34(18.7)	44(24.2)	1.632^a^	0.125
Alcohol, *n* (%)	21(11.5)	13(7.1)	2.076^a^	0.103
Smoking, *n* (%)	29(15.9)	33(18.1)	0.311^a^	0.338
Heartburn, *M* (Q₁, Q₃)	1.0(1.0,2.0)	1.50(1.0,2.0)	−0.384^b^	0.701
Regurgitation, *M* (Q₁, Q₃)	1.0(0.0,2.0)	1.00(1.0,3.0)	−1.853^b^	0.064
Clinical charactistics				
Bloating, *n* (%)	74(40.7)	98(53.8)	6.349^a^	0.008
Belching, *n* (%)	64(35.2)	63(34.6)	0.012^a^	0.500
Inappetence, *n* (%)	68(37.4)	63(34.6)	0.298^a^	0.331

^a^Chi-square Test, ^b^Mann–Whitney *U* test, ^c^Gastroesophageal reflux disease, ^d^*Helicobacter pylori*.

### 3.3 Correlation of GMI and the aortomesenteric angle with different grades of gastroptosis

**Figure 3:**
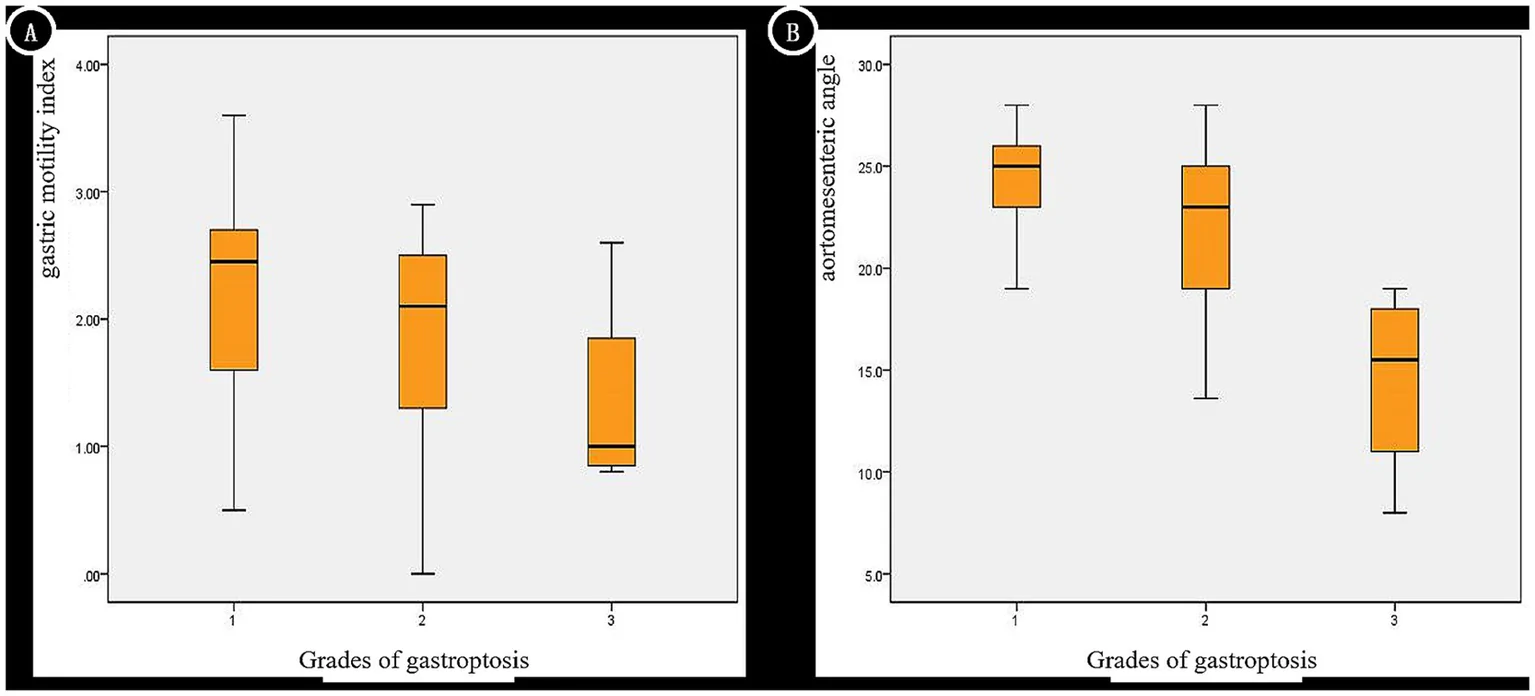
Box plot of GMI and aortomesenteric angle for patients with different grades of gastroptosis: **(A)** GMI in patients with different grades of gastroptosis, **(B)** aortomesenteric angle in patients with different grades of gastroptosis.

The Kruskal–Wallis *H* test was applied to the gastric contrast-enhanced ultrasound parameters of patients with different grades of gastroptosis after matching. The results demonstrated statistically significant differences among the three grades in terms of GMI and aortomesenteric angle (*p* < 0.05) (Table 3 and Figure 3).

**Table 3:** Comparison of GMI and aortomesenteric angle in patients with different grades of gastroptosis.

Variables	I	II	III	Test Statistic	*P*
GMI, M (Q₁, Q₃)	2.45(1.50,2.70)	2.1(1.27,2.50)	1.0(0.83,2.23)	10.438^c^	0.005
Aortomesenteric angle, M (Q₁, Q₃)	25(23,27)	23(19,25)	16(10,22)	26.042^c^	<0.001

^c^Kruskal–Wallis *H* Test.

Variables that showed a statistically significant difference between groups in Table 1 were entered as independent variables into a multivariate binary logistic regression analysis, with the final diagnosis of gastroptosis as the dependent variable. The forward selection method was used for variable entry. The results showed that BMI, GMI, and aortomesenteric angle were independent influencing factors for gastroptosis after matching (*p* < 0.05) (Table 4).

**Table 4:** Binary logistic regression analysis after propensity score matching.

Variables	*β*	SE	Wald *χ*^2^	*P*	OR (95%CI)
BMI	−0.413	0.076	29.300	<0.001	0.661 (0.570 ~ 0.768)
GMI	−1.972	0.282	48.738	<0.001	0.139 (0.080 ~ 0.242)
Aortomesenteric angle	−0.147	0.033	19.750	<0.001	0.863 (0.809 ~ 0.921)
Duodenal reflux	0.265	0.347	0.581	0.446	1.303 (0.660 ~ 2.573)
Bloating	0.092	0.315	0.084	0.772	1.096 (0.591 ~ 2.033)

### 3.4 ROC curve analysis

**Figure 4:**
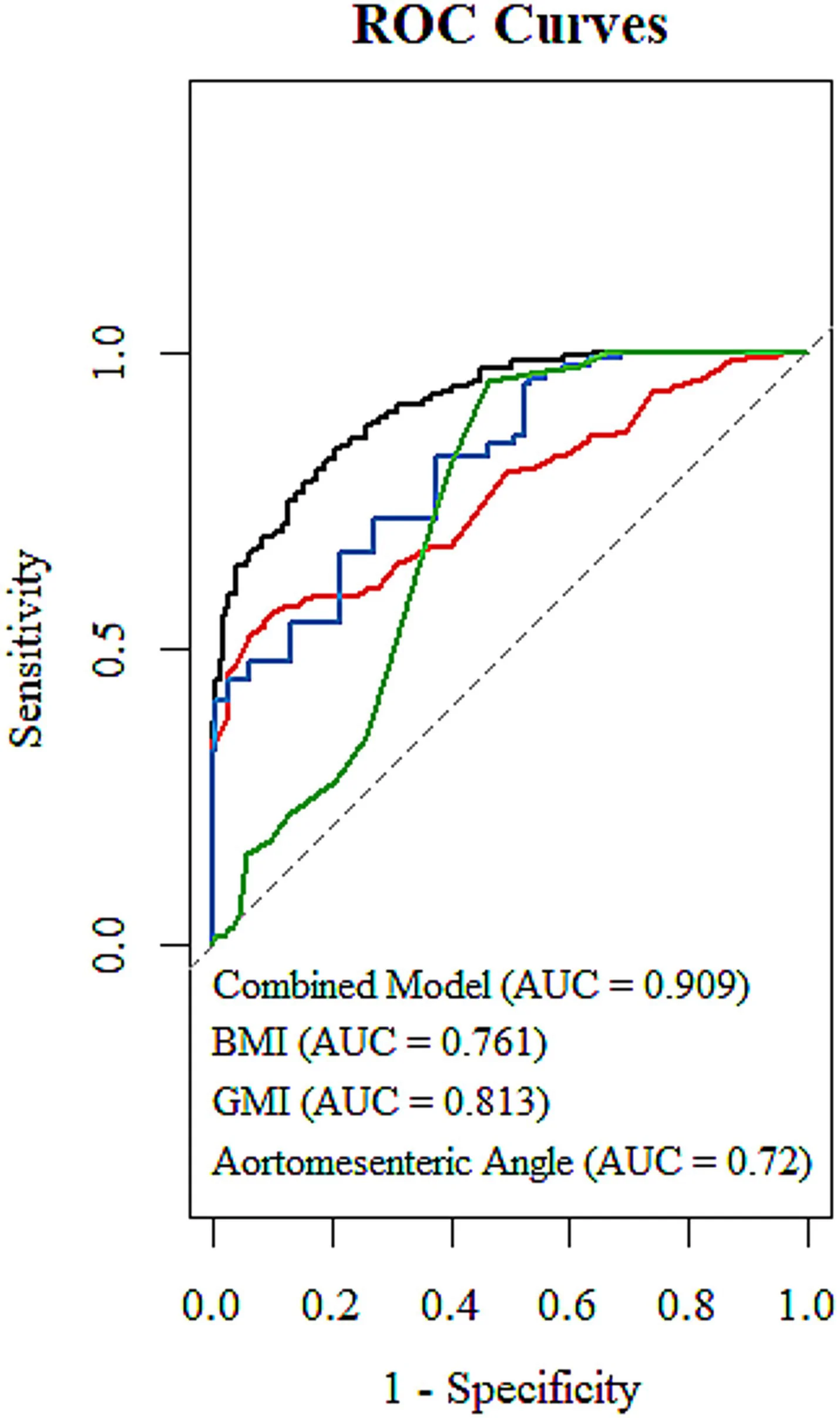
ROC curve analysis of positive variables based on multiple factor analysis.

The ROC curve analysis was performed to evaluate the predictive value of BMI, GMI, and the aortomesenteric angle for gastroptosis. The AUC for BMI, GMI, and the aortomesenteric angle alone were 0.761, 0.813, and 0.720, respectively. GMI showed the highest prediction accuracy (AUC: 0.813, sensitivity: 82%, specificity: 63%, Youden index: 45%). The combined model yielded an AUC of 0.909, with a sensitivity of 0.84, a specificity of 0.80, and a Youden index of 0.63, indicating good discriminative ability for identifying gastroptosis (Figure 4 and Table 5).

**Table 5:** The AUC, cut-off value, sensitivity, specificity, and Youden index for BMI, GMI, the aortomesenteric angle, and the combined predictor.

Variables	AUC	Cut-off Value	Sensitivity	Specificity	Youden index
BMI	0.761	20.85	0.56	0.9	0.46
GMI	0.813	2.71	0.82	0.63	0.45
Aortomesenteric angle	0.720	28.5	0.95	0.54	0.49
Combined predictor	0.909	0.44	0.84	0.80	0.63

## 4 Discussion

Currently, there is no consensus regarding the baseline age of onset for gastroptosis. Although existing research predominantly focuses on pediatric and young adult cohorts, this does not necessarily indicate a higher prevalence in younger populations (6–8), as reported cases span a wide age distribution. Consequently, a systematic investigation into the clinical characteristics of gastroptosis across different age groups is warranted. Furthermore, while some studies suggest a higher prevalence in young women, these findings are of limited contemporary relevance and generalizability due to their historical nature (32).

Based on the PSM analysis comparing patients with and without gastroptosis, we found that BMI was inversely associated with the odds of gastroptosis, identifying a low BMI as an independent risk factor for the condition. This finding aligns with the results reported by Van et al. (8). As BMI is a key indicator of nutritional status, it is noteworthy that the majority of gastroptosis patients in our cohort exhibited varying degrees of malnutrition. Impaired nutritional status likely reduces overall adipose tissue, thereby compromising the tensile strength and tone of the supporting ligaments and mesentery that maintain the normal anatomical position of the stomach. Consequently, this structural weakening facilitates downward gastric displacement (3, 33).

The GMI serves as an objective tool to assess both the severity of gastroptosis and its therapeutic response, as OGUS effectively visualizes pyloric motor activity and antral motility (34). In this study, we observed a significant difference in GMI between patients with and without gastroptosis, with GMI being inversely associated with disease occurrence. A low GMI was identified as an independent risk factor for gastroptosis and correlated with its severity, suggesting that these patients experience varying degrees of gastric emptying dysfunction. This dysfunction likely underpins their susceptibility to symptoms such as bloating, belching, and acid reflux (35). Furthermore, the incidence of duodenal reflux differed significantly between the two groups. Typically associated with vagal nerve dysfunction and impaired pyloric sphincter tone, duodenal reflux involves the retrograde transit of duodenal chyme into the stomach. Concurrently, delayed gastric emptying prolongs the retention of gastric contents. Under this dual pathology, the movement of luminal contents between the stomach and duodenum exhibits a ‘reciprocal flow’ phenomenon. This not only elevates intragastric pressure but also allows bile, pancreatic enzymes, and intestinal secretions to enter the stomach, thereby exacerbating mucosal damage (36–38). This gastric emptying impairment is further supported by physiological evidence suggesting that chronic oxidative stress injury to the gastric wall depletes gastric pacemaker cells—specifically the interstitial cells of Cajal—disrupting the gastric slow-wave rhythm (39, 40).

Previous studies have reported that gastroptosis patients primarily present with symptoms such as nausea, vomiting, abdominal distension, and early satiety, which aligns with our observations (41, 42). Concurrently, when comparing symptoms between patients with and without gastroptosis, we noted that a substantial proportion of gastroptosis patients experienced a loss of appetite. This may be attributed to impaired gastric motility and prolonged gastric emptying times, which in turn exacerbate early satiety.

Although the clinical utility of the GerdQ score has been increasingly constrained by its inherent subjectivity—especially with the emergence of objective diagnostic modalities such as 24-h ambulatory pH monitoring, esophageal manometry, and rapid salivary pepsin assays—it remains a valuable tool for rapid outpatient screening of reflux-related symptoms (41, 42). Therefore, to minimize the potential confounding effects exerted by other items within the GerdQ score, we specifically analyzed the correlation of items Q1 and Q2 with gastroptosis. Notably, no statistically significant differences were observed between the two groups regarding the incidence of heartburn and regurgitation, nor in the overall detection rates of GERD. This finding aligns consistently with the previous study by Kusano et al. (32). This further supports the limitation of GerdQ score in definitively diagnosing GERD.

Indeed, the pathological link between gastroptosis and GERD remains controversial. Mechanistically, the caudal descent of the gastric body and antrum can trigger transient lower esophageal sphincter relaxations, facilitating the reflux of chyme accumulated in the gastric fundus into the esophagus and increasing GERD risk. Paradoxically, however, as gastroptosis severity increases, further elongation of the gastric body and expanded gastric capacity may actually reduce gravity-induced reflux episodes (32). Instead, gastroptosis appears more closely associated with alkaline reflux, potentially driven by pyloric and duodenal dysmotility that promotes the retrograde flow of bile and other duodenal contents. Nevertheless, the precise pathophysiological mechanisms coupling gastroptosis with alkaline reflux remain to be fully elucidated (6, 45).

**Figure 5:**
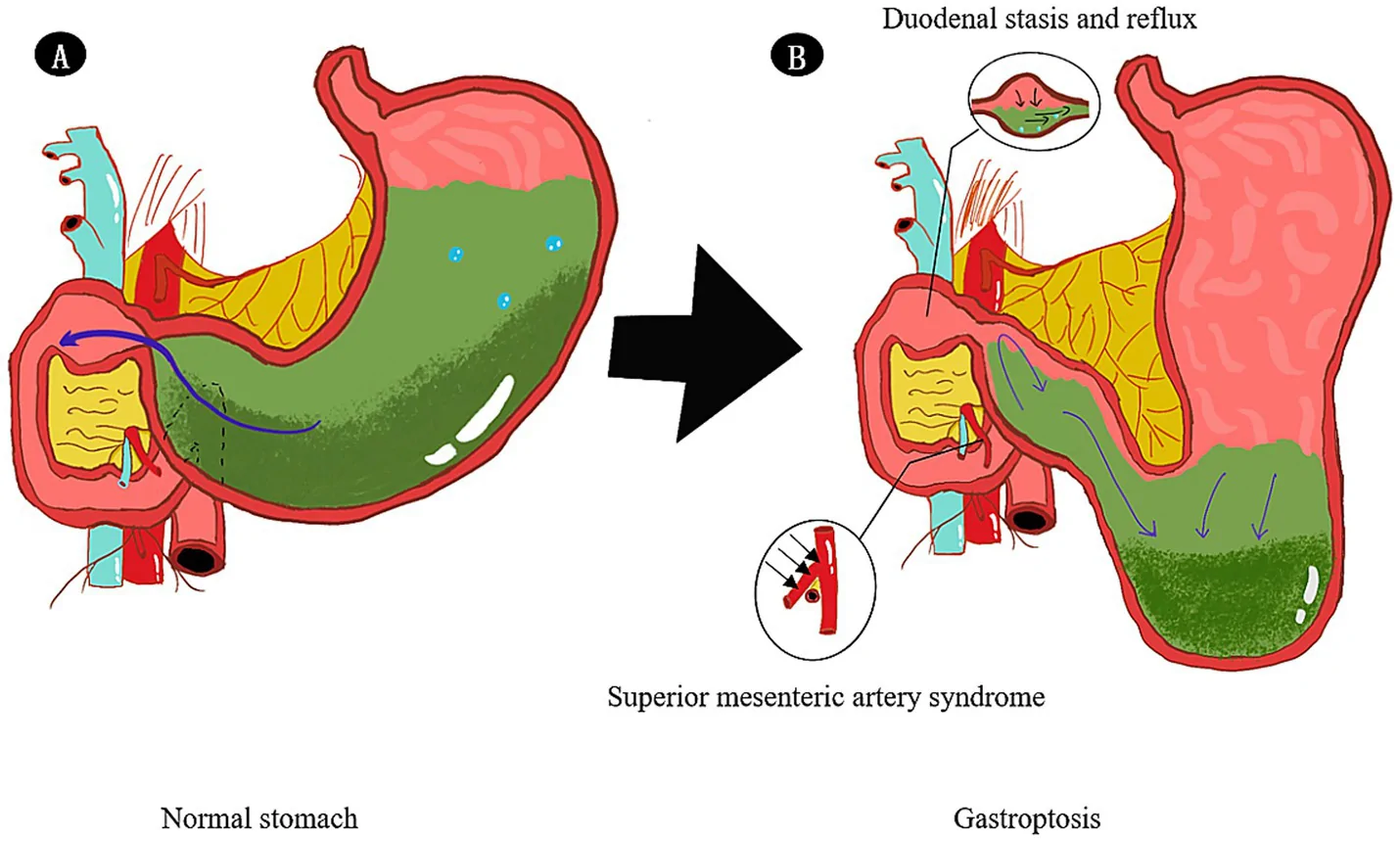
Gastric emptying disorders in gastroptosis combined with SMAS: **(A)** It shows the normal stomach when the chyme can smoothly pass through the pylorus. **(B)** It shows mechanical intestinal obstruction and gastroptosis ensues when the duodenum is long compressed by the superior mesenteric artery, resulting in duodenal stasis and reflux.

Our findings indicate that the aortomesenteric angle is a key determinant in the development of gastroptosis. SMAS is an anatomical disorder characterized by an abnormally narrow aortomesenteric angle, which compresses the third and transverse portions of the duodenum, leading to acute or chronic proximal duodenal obstruction. Notably, the clinical presentation of SMAS—including abdominal distension, pain, and vomiting—closely mirrors that of gastroptosis (46, 47). Youssoufi et al. (48) suggested that long-standing SMAS can contribute to duodenal stenosis. When gastroptosis coexists with SMAS, patients often present with profound, sustained weight loss and severe gastric emptying disorders; chronic nausea, vomiting, and dyspepsia represent the synergistic consequences of both pathologies (Figure 5). Although direct evidence linking SMAS-induced duodenal obstruction to gastroptosis remains scarce in the current literature, the shared clinical features of decreased gastric motility, malnutrition, and abdominal symptoms suggest overlapping pathophysiological pathways in their development. Consequently, further multicenter, prospective studies are warranted to fully elucidate the pathophysiological relationship between SMAS and gastroptosis (49–51).

This study has several limitations. (1) There are currently no established clinical consensus guidelines for the diagnosis of gastroptosis in China. The diagnostic criteria utilized herein were adapted from the 1959 Japanese Society of Radiology guidelines (9). While this historical classification remains one of the few documented radiographic frameworks for grading gastroptosis, its chronological gap and potential confounding by ethnic, regional, and lifestyle variations may introduce diagnostic inaccuracies when applied to a modern Chinese population. This highlights an urgent need to develop updated diagnostic standards aligned with contemporary clinical practice in China. (2) As a single-center retrospective study, despite utilizing PSM to minimize selection bias, we could not completely eliminate confounding from unmeasured variables; thus, causal relationships cannot be definitively established. (3) The accuracy of oral contrast-enhanced gastric ultrasonography is inherently subject to intragastric gas interference and inter-operator variability, which may introduce measurement errors in gastric motility and compromise diagnostic reliability. (4) Gas interference also precluded standardized measurements of the inner diameter of the transverse duodenum, limiting our evaluation of duodenal peristalsis and dilation to qualitative descriptions. (5) The diagnostic utility of the aortomesenteric angle in gastroptosis lacks validation in existing literature, warranting further investigation to confirm its clinical significance. (6) Several diagnostic and grading criteria utilized in this field currently lack authoritative consensus in the literature. These include the radiographic and contrast-enhanced ultrasonographic criteria for grading gastroptosis severity, the standardized thresholds for assessing gastric motility via GMI, and the diagnostic parameters for pathological duodenal reflux. Establishing and validating these criteria will be a primary focus of future research to better characterize the imaging and clinical features of gastroptosis. (7) Given the paucity of contemporary clinical research on gastroptosis, large-scale prospective cohort studies are required to validate our findings.

## 5 Conclusion

In conclusion, a low BMI, reduced GMI, and a narrowed aortomesenteric angle are independently associated with gastroptosis. Clinically, early symptoms of gastroptosis typically include abdominal bloating, early satiety, and appetite loss. Without timely intervention, these symptoms may progress to belching and vomiting, while patients with severe gastroptosis may present with clinical manifestations of malnutrition, such as weight loss and anemia. Consequently, these parameters may serve as valuable clinical indicators for early detection. Most notably, future prospective multicenter studies are warranted to validate these findings, which holds significant potential for optimizing early clinical intervention and prevention strategies.

## Data Availability

The raw data supporting the conclusions of this article will be made available by the authors, without undue reservation.
