# Supplementary material for: Clinical characteristics and early recognition of gastroptosis: a propensity score-matching analysis
Source: Front Med (Lausanne). 2026 Aug 19;13:1916142. doi: 10.3389/fmed.2026.1916142 (PMC13533760; doi:10.3389/fmed.2026.1916142)
Supplement: Supplementary file 1 [file Data_sheet_1.pdf]

Supplementary Table 1. GerdQ questionnaire

Question	Frequency score for symptom			
	0 days	1 days	2-3 days	4-7 days
1. How often did you have a burning feeling behind your breastbone (heartburn)?	0	1	2	3
2. How often did you have stomach contents (liquid or food) moving upwards to your throat or mouth (regurgitation)?	0	1	2	3
3. How often did you have a pain in the centre of the upper stomach?	3	2	1	0
4. How often did you have nausea?	3	2	1	0
5. How often did you have difficulty getting a good night's sleep because of your heartburn and ⁄ or regurgitation?	0	1	2	3
6. How often did you take additional medication for your heartburn and ⁄ or regurgitation, other than what the physician told you to take? (such as Tums, Rolaids, Maalox?)	0	1	2	3
